# First report of *Neoscytalidium dimidiatum* as the causal agent of leaf blight on *Clivia miniata*

**DOI:** 10.1038/s41598-023-43144-4

**Published:** 2023-09-26

**Authors:** Zahra Zaeimian, Khalil-Berdi Fotouhifar

**Affiliations:** Department of Plant Protection, Faculty of Agriculture, College of Agriculture and Natural Resources, University of Tehran, Karaj, 31587-77871 Iran

**Keywords:** Molecular biology, Plant sciences

## Abstract

In this survey, the symptomatic leaves of *Clivia miniata* were collected from a greenhouse in Karaj city of Iran. The isolation and morphological investigation showed *Scytalidium*-like fungus associated with leaf blight symptom. The phylogenetic analysis of the internal transcribed spacer along with partial sequences of rDNA large subunit and translation elongation factor 1-α (*tef*-1α) genomic regions confirmed the identification of the recovered isolate as *Neoscytalidium dimidiatum*. The pycnidial morph of the fungus didn’t observe both in vitro and in vivo. The pathogenicity test on *C. miniata* and *C. nobilis* was also conducted to fulfill the Koch’s postulates. To our Knowledges, this is the first report of *N. dimidiatum* causing leaf blight disease on *C. miniata* and *C. nobilis* worldwide, as well as these host plants are new for *N. dimidiatum* in the world.

## Introduction

The genus *Neoscytalidium* belongs to the Botryosphaeriaceae family (Ascomycota), produces two types of anamorphs which are known as arthric with *Scytalidium*-like conidia and pycnidial that produces *Fusicoccum*-like conidia^1^. The arthric and pycnidial states were named *Torula dimidiata* and *Hendersonula toruloidea* respectively^1^. Later, *Scytalidium hyalinum* was introduced, which has the arthric mycelium^2^. *Hendersonula toruloidea* was revised and transferred into a new genus, *Nattrassia*, with *N. mangiferae* as the type species. The *Scytalidium*-like synanamorph was named as *Scytalidium dimidiatum*^3^. During a phylogenetic revision of the Botryosphaeriaceae family, polyphyletic nature of the genus *Scytalidium* was revealed, and *Neoscytalidium* was introduced as a new genus and *S. dimidiatum* was considered as a synonym for *N. dimidiatum*^4^. *Neoscytalidium novaehollandiae* was introduced as new species based on the production of muriform and *Dichomera*-like conidia, differing from *N. dimidiatum*^5^. *Scytalidium hyalinum* has been identified as a melanin-deficient mutant of *S. dimidiatum*, and introduced as *N. dimidiatum* var. *hyalinum*^3,6^. The *N. hyalinum* was chosen as the type species of *Neoscytalidium,* instead of *N. dimidiatum*^2^. Considering the fact that “*dimidiatum*” (1882) is an older epithet than “*hyalinum*” (1977), it was proposed that the correct type species is *N. dimidiatum. Neoscytalidium orchidacearum* was introduced as a new species only on orchids in Thailand, up to now. It has been mentioned that the fungus isolated as *N. dimidiatum* from rhinosinusitis in Iran, may be another new species^3,7^. *Neoscytalidium oculus* was reported as a new species based on the absence of a coelomycetous synanamorph from keratitis^8^. Until 2020, it was believed that *Neoscytalidium* has four species including *N. dimidiatum*, *N. novaehollandiae*, *N. orchidacearum*, and *N. oculus*^9^. The similarity of the ex-type sequence of *N. dimidiatum*, *N. novaehollandiae*, and *N. orchidacearum* was confirmed based on the ITS, *tef1* and *tub2* genomic regions. *Neoscytalidium novaehollandiae* and *N. orchidacearum* were considered as synonyms for *N. dimidiatum*, and it was mentioned that the *N. dimidiatum* is the only species in the genus *Neoscytalidium*^10^. This suggestion was confirmed by the phylogeny of the ITS, LSU, *chs-1* and *tub2* genomic regions^11^. Recently, *N. hylocereum* was reported as a new species causing canker on *Hylocereus polyrhizus* in Thailand^12^.

*Neoscytalidium* species can be plant pathogens, endophytes, saprobes, and latent pathogens. It is also considered as a mycosis agent in human and animals^2,6,9,13^. This genus distributed all over the world except Arctic and Antarctic areas^2^. Although, they are almost observed on woody plant hosts, but they can be found on leafy plants, soils, and mammals^6,8,13,14^. *Neoscytalidium dimidiatum* is an opportunistic pathogen and can cause veriety of diseases in plants^1,2,6,9,13,15–24^ as well as in human^3,6–8,11,13–15,25^.

In Iran, *Neoscytalidium dimidiatum* has been isolated from pistachio trees as one of the causal agents of young shoots dieback in Kerman province^26^. It has also been reported as a causal agent of Citrus branch wilt, death and decline disease in Khouzestan province^27^. The *N. mangiferae* was reported as the causal agent of dieback and trunk cankers on *Ficus religiosa* and dieback on *Psidium guajava* in Jiroft City, Kerman province^28^. It has also been isolated from *Acer*, *Platanus*, *Magnolia*, *Eryobotria*, *Eryobotria*, *Morus*, *Ulmus*, *Cupressus*, *Robinia*, *Eucalyptus*, *Citrus*, *Juglans* and *Malus* as a causal agent of decline disease in Shiraz City, Fars province^29^. *Neoscytalidium hyalinum* has been isolated from wood lesion and decline symptoms of willow (*Salix* spp.) and poplar (*Populus* spp.) in Shiraz City, Fars province^30^. It has been reported as a associated fungus with trunk disease on *Calligonum amoenum* in Kerman province^31^. Also, this species was isolated from dieback and canker symptoms of the date palm in Fars, Kerman, Khouzestan, Hormozgan, Isfahan, and Sistan and Balochestan provinces^32^. *Neoscytalidium dimidiatum* has also been reported as a pathogen associated with Persian oak decline in Ilam province^33^. *Neoscytalidium hyalinum* has been reported causing fruit rot on *Cucumis melo* in Fars province^34^. This fungus has also been isolated from neem tree (*Azadirachta indica*) decline symptom in Hormozgan province^35^. *Neoscytalidium dimidiatum* has been reported as a associated fungus with walnut decline in Kerman, Kermanshah, Hamedan, Kurdistan, and Isfahan provinces^36^. This species has also been reported as a associated fungus of sooty canker and dieback on *Ficus benghalensis* in Kish Island, Hormozgan province^37^. Also the fungus has recently been isolated as a associated fungus with necrotic wood tissues of pomegranate in Kerman and Fars provinces^38^.

The genus *Clivia* (Amaryllidaceae) is an important herbaceous plant that are grown worldwide for cut-flower trade^39,40^. *Clivia miniata* (Lindl.) Verschaff., is the most well-known species, known as Natal lily, bush lily, Kaffir lily, Boslelie, or Umayime in different regions^39^. Also, the medicinal, pharmacological, and phytochemical features of this species have been studied^39,41^.

Fungal diseases of the *Clivia* spp. have been investigated many times in the world. *Stagnospora curtisii* has been reported as a associated fungus of leaf and stalk spot of *Clivia* spp. in New Zealand^42^. *Colletotrichum gloeosporiodes* has been reported causing anthracnose disease on *C. miniata* in South Africa^43^. *Colletotrichum trichellum* has also been reported causing anthracnose on *C. miniata* in Korea^44^. *Macrophoma agapanthii* as a causal agent of leaf dieback, and *Sclerotium rolfsii* as a causal agent of collar rot on *Clivia* spp. have been reported in South Aferica^45^. *Colletotrichum boninense* has been reported as a causal agent of anthracnose on *C. miniata* in Japan, and later the name of the species has been changed to *C. karsti*^46,47^. *Fusicoccum luteum* has been identified causing leaf spot and plant collapse in *C. miniata* in Northland^48^. *Colletotrichum cliviae* has been reported as a causal agent of anthracnose on *C. miniata* in China, and later the name of the species has been changed to *C. cliviicola*^49,50^. *Alternaria tenuissima*, *Blennoria* sp., *Phoma* sp., and *Strelitziana cliviae* have been reported on *Clivia* as plant pathogens^40^. *Fusarium solani* and *F. proliferatum* have been identified as the causal agents of root rot on *C. miniata* in China^51^. *Fusarium proliferatum* has been reported causing sheath rot on *C. miniata* in China^52^. *Fusarium oxysporum* has also been reported as the causal agent of basal stem rot on *C. miniata* in China^53^. *Fusarium solani* has been reported causing sheath rot on *C. miniata* in China^41^. *Botrytis cinerea* (China), *C. himantophylli* (Canada), *Gloeosporium crini* (Korea), *Gloeosporium* sp. (China), *Myrothecium leucotrichum* (Florida), *Pestalotia glandicola* (Japan), and *Physalospora cliviae* (Southern Africa) have also been reported on *Clivia*^54^. *Colletotricum trichellum* has been reported causing anthracnose on *C. miniata* in Tehran province, in Iran^55^.

According to the importance of *Clivia* species conservation from extinction^39^, identification of the biotic infectious agents, especially fungi, is very important. Despite the economic importance of *Clivia* spp. in Iran, there is only one study regarding the diseases of this plant. So, the main aim of the present study was the isolation and morphological and molecular characterization of the causal agent of the leaf blight disease on *C. miniata* and fulfilling the Koch’s postulates to confirm the pathogenicity of the recovered fungal agent.

## Materials and methods

### Sampling and isolation

The *C. miniata* leaves with leaf blight symptom were collected from a greenhouse of the Department of Horticulture, College of Agriculture and Natural resources, University of Tehran, Karaj (N35° 48′ 20.4″ E 050° 59′ 53.9″) according to the guidelines of the University of Tehran. The leaf samples were surface sterilized by 70% ethanol. The boundaries of the diseased and healthy parts of the leaf samples were cut into 1 $\times$ 1 cm small pieces, submerged in 70% ethanol, and then in 2% sodium hypochlorite, each step for 2 min. The leaf pieces were washed three times with distilled water, and then were transferred onto the sterile paper towel to dry in room temperature. The specimens were placed onto the 1.7% water agar culture medium and incubated at 26 ℃ in dark condition for seven days. When the mycelia grow from the leaf pieces, they were purified using the hyphal tip method, and the hyphal tips were transferred onto the 2% potato dextrose agar culture medium and incubated at the same above mentioned conditions.

### Morphological identification

As the fungal colony grows, its diameter was measured until it filled the nine cm in diameter Petri dish. The surface and reverse colour of the five-days and 15-day old colony was recorded according to the Rayner’s colour chart^56^. The microscopic slide mounts were prepared from 15-day old colony using the lactophenol, and in the microscopic slides, the mycelium and spore features were examined under the olympus BH2 light microscope. In order to fruit body formation in the recovered fungal isolate, the isolate was grown on 2% pine needle agar (PNA) culture medium (2% water agar with double sterilized pine needle in autoclave) and the inoculated Petri dishes were kept under the 12 h near UV light/12 h dark condition at 25 ℃, and the colony was inspected every day until the 30 days. Morphological identification of the recovered isolate was done based on the description provided by Crous et al*.*^4^.

### Molecular identification

#### DNA extraction

The modified Zhong and Steffenson’s^57^ protocol was used to extract the genomic DNA from the recovered fungal isolate. In brief, 10–20 g of mycelium was harvested from the four-day old colony on PDA culture medium, and the lysis buffer was added and the mycelia were ground very well. The mixture was transferred to a sterile microtube and vortexed. The microtube was placed on the 65 ℃ hot-plate for 15 min and inverted every five minutes. Then, it was placed on the ice and kept at − 20 ℃ for 10 min. In order to remove proteins, chloroform:isoamyl alcohol (24:1) was added and kept in the same above mentioned condition. The mixture was centrifuged at 10,000 rpm for 10 min. The supernatant was transferred to a new microtube, chloroform was added and the mixture was centrifuged again. The supernatant was transferred to a new microtube and equal volume of isopropanol was added. Three phases were appeared in the mixture, where the middle phase containing the nucleic acid. The mixture was inverted, centrifuged at 12,000 rpm for 10 min, and the supernatant was discarded. The 70% ethanol was added and the mixture was centrifuged at 10,000 rpm for three minutes. After removing the supernatant, the microtube was placed upside-down on the 65 ℃ hot-plate for 30 min. Finally, 30 µl deionized sterile water was added and spined shortly. The extracted DNA was kept at 4 ℃ overnight, and then stored at -20 ℃ for future use. The success of the DNA extraction was investigated using the electrophoresis in 1% agarose gel and also by NanoDropping.

### Polymerase chain reaction and sequencing

The internal transcribed spacer (ITS), partial sequence of large subunit (LSU), and partial sequence of translation elongation factor 1-α (*tef1-α*) genomic regions were amplified using the ITS1 (5̍-TCCGTAGGTGAACCTGCGG-3̍) and ITS4 (5̍-TCCTCCGCTTATTGATATGC-3′), LROR (5′-ACCCGCTGAACTTAAGC-3′) and LR5 (5′-ATCCTGAGGGAAACTTC-3′), EF1-728F (5′-CATCGAGAAGTTCGAGAAGG-3′) and EF1-986R (5′-TACTTGAAGGAACCCTTACC-3′) primer pairs, respectively^58–60^. The PCR mixture (25 µl total volume) consisted of 12.5 µl PCR Mastermix, 8.5 µl deionized water, 0.5 µl for ITS and 1 µl for LSU and *tef1-α* of primers (10 pmol), 3 µl (for ITS) and 2 µl of 30 ng/µl extracted DNA (for LSU and *tef1-α*). The PCR condition was as follow for ITS, LSU, and *tef1-α*, respectively: initial denaturation (95 ℃ for 90s, 95 ℃ for 2min, 95 ℃ for 5min), 35 cycles of denaturation (94 ℃ for 30s, 95 ℃ for 30s, 94 ℃ for 50s), annealing (52 ℃ for 30s, 51 ℃ for 45s, 56 ℃ for 45s), and extension (72 ℃ for 30s, 72 ℃ for 1min, 72 ℃ for 1min), and with the final extension (72 ℃ for 6min, 72 ℃ for 8min, 72 ℃ for 10min)^61^. The accuracy of the PCR products was evaluated by electrophoresis in 1% agarose gel and the PCR products of ITS and both of LSU and *tef1-α* genomic regions were sent for sequencing to Bio-Magic-Gene International Company and Takapouzist (Iran), respectively.

### Phylogeny analysis

The obtained sequences were deposited in the National Center for Biotechnology Information (NCBI) (https://www.ncbi.nlm.nih.gov/) and also these sequences were subjected to BLAST search in NCBI database using the Basic Local Alignment Search Tool (BLAST) (https://blast.ncbi.nlm.nih.gov/). The closest related sequences of the species and genera to these queries were obtained from GenBank, and all sequences were edited by Chromas software version 2.6.6. The sequences of three genomic regions were concatenated in each isolate. The datasets were aligned with MUSCLE program of the MEGA version 11 software using the default settings. The best model for generating maximum likelihood phylogenetic tree was determined by MEGA software using all sites of gaps or missing data treatment as the default setting. The maximum likelihood and maximum parsimony phylogenetic trees were constructed for ITS-TEF, ITS-LSU and ITS-LSU-TEF concatenated sequences. For generating each tree, the bootstrap method with 1000 replications was used. The all sites of gaps or missing data were also considered in the tree reconstruction. Finally, the phylogenetic trees were built in MEGA version 11 software and their topologies were compared.

### Pathogenicity test

The Koch’s postulates were conducted to confirm the pathogenicity of the recovered isolate. For each *Clivia* species, one pot having one plant was used. For pathogenicity test, 20cm in diameter and 18cm in height plastic pots were used. Each pot had one three-year old growing and healthy plant that was propagated offset. Three replicates were used in the treatment as well as in the control. The soil composition of the pots was a mixture of garden soil, sand, and rotted leaf soil that each of them made a third of the mixture. The surface of the four healthy leaves of each plant in each pot was disinfected with 70% ethanol^13^. In each leaf, four points were wounded using a sterile 6 mm in diameter cork borer. The mycelial plugs (6 mm in diameter) were taken from the margin of the seven-day old colony and placed upside-down on the created wounds and wrapped with cellophane. The mycelium free PDA plugs were used for the inoculation of the wounds in control plants. Each pot was covered by black plastic bag for three days to provide dark condition and preserve the moisture, and after three days the plastic bag was removed. The pots were irrigated every two days. The tests were monitored daily, until the symptom of the disease appeared. The experiment was performed twice on *C. miniata* and *C. nobilis*. Furthermore, the experiment was conducted in two different temperature ranging 26–28 ℃ and 30–32 ℃, to investigate the effect of this factor on the pathogenicity of the recovered fungus.

## Results

**Figure 1:**
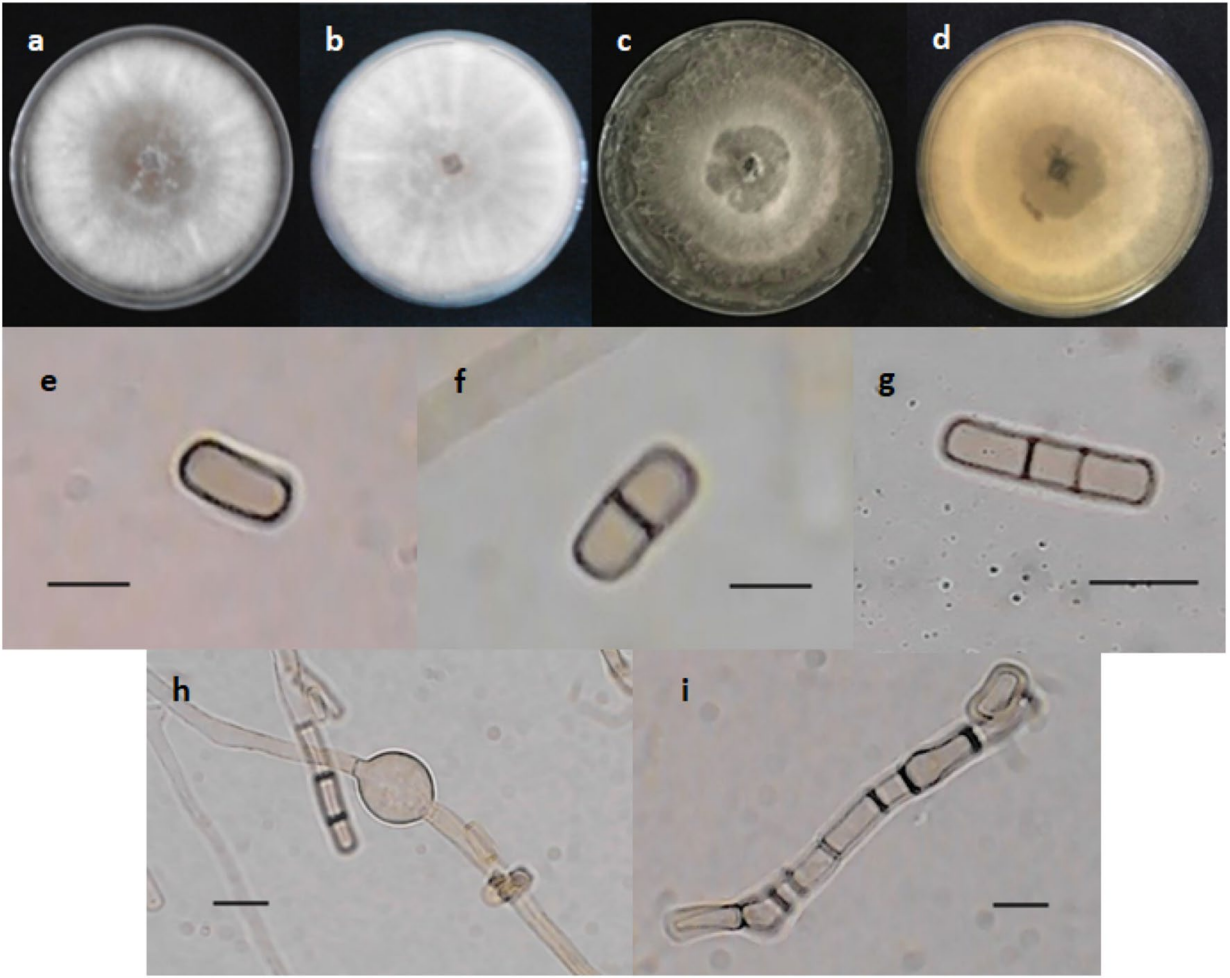
Morphological features of the *Neoscytalidium dimidiatum*, isolate GKH-2. (**a** and **b**) Surface and reverse of five-day old colony; (**c** and **d**) Surface and reverse of the 15-day old colony; (**e**–**g**) Aseptate to 2-septate arthroconidia; (**h**) Swelling in hypha; and (**i**) Arthric chain. Scale bars: e and f = 6µm; g = 8 µm; h and i = 4 µm.

The obtained axenic isolate filled the nine cm in diameter Petri dish after five days. The average growth rate of the isolate was 1.77 cm/day. The surface colour of the five-day old colony was white becoming pale mouse grey at the center and it was white on the reverse. The colony colour was olivaceous grey and buff for the surface and the reverse of the 15-day old colony, respectively. The isolate produced powdery to the touch aerial mycelia and *Scytalidium*-like arthroconidia. The conidia were various in shape including globose to subglobose, ellipsoidal, cylindrical, oblong, obtuse, truncate, and articulated from the mycelia as singular or in chain. They were hyaline to pale brown, thick walled, 0–2 septate, and 4–28 (9.85) $\times$ 2.5–5 (7.04) µm in size (n = 50). The hyphae rarely had swellings (Fig. 1). The arthroconidia were aggregated on the pine needle on PNA culture medium, but the differentiation of the hyphae and formation of the pycnidia did not occur. These morphological characteristics of the recovered isolate were similar to the genus *Neoscytalidium* according to the Crous et al.^4^ description.

**Figure 2:**
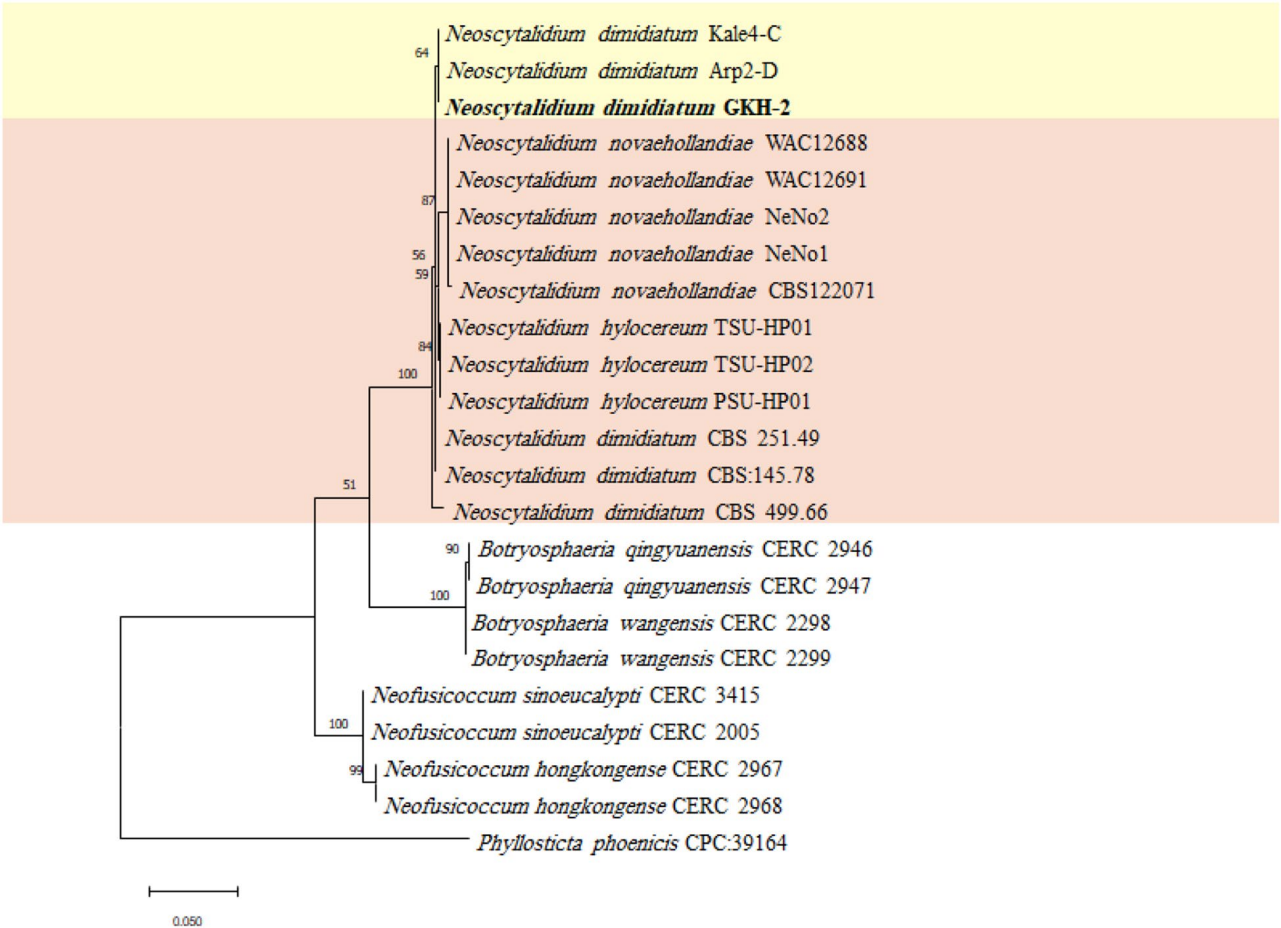
Maximum likelihood phylogenetic tree of the concatenated ITS and *tef1-α* sequences, generated by MEGA ver. 11 software. The suggested T92 + I model by MEGA software was used. *Phyllosticta phoenicis* was used as an outgroup taxon. The recovered fungal isolate in the present study was shown in boldface. The bootstrap values more than 50% have been presented on the branches.

**Figure 3:**
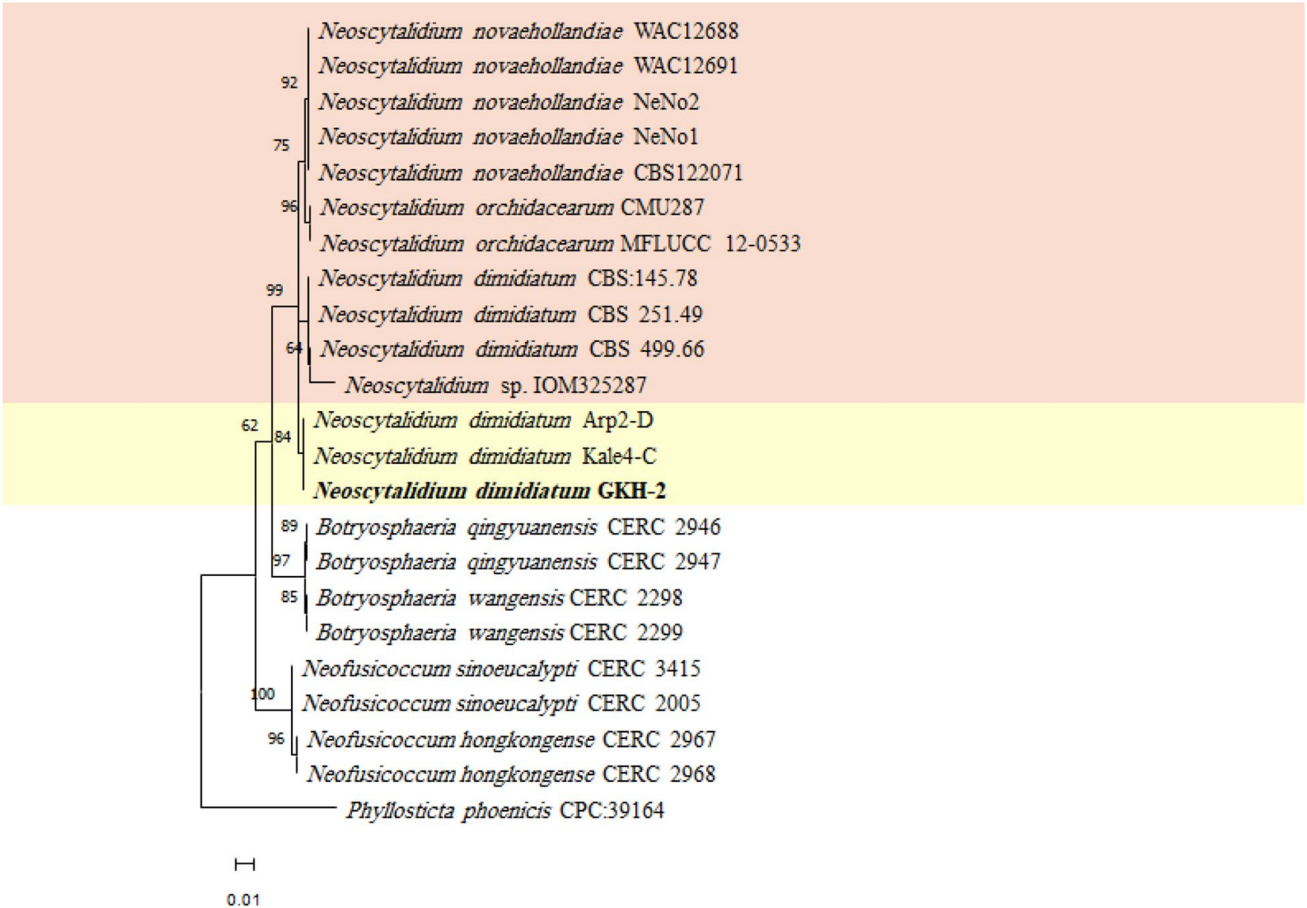
Maximum likelihood phylogenetic tree of the concatenated ITS and LSU sequences, generated by MEGA ver. 11 software. The suggested K2 + G model by MEGA software was used. *Phyllosticta phoenicis* was used as an outgroup taxon. The recovered fungal isolate in the present study was shown in boldface. The bootstrap values more than 50% have been presented on the branches.

**Figure 4:**
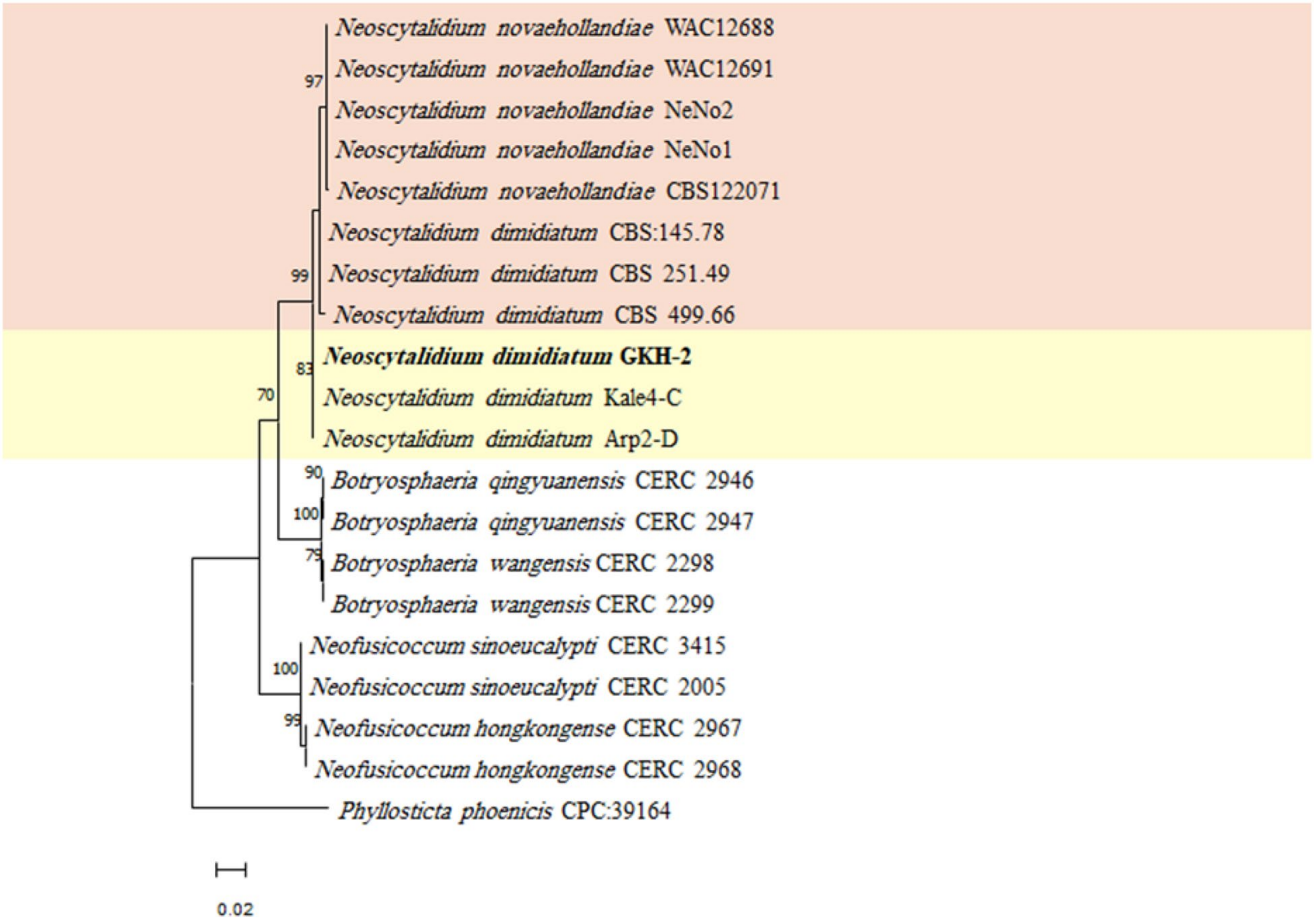
Maximum likelihood phylogenetic tree of the concatenated ITS, LSU and *tef1-α* sequences, generated by MEGA ver. 11 software. The suggested T92 + G model by MEGA software was used. *Phyllosticta phoenicis* was used as an outgroup taxon. The recovered fungal isolate in the present study was shown in boldface. The bootstrap values more than 50% have been presented on the branches.

The PCR amplified 554, 916, and 297 bp fragments from ITS, LSU, and *tef1-α* genomic regions, respectively. These sequences were deposited in the GenBank (NCBI) with accession numbers of MZ047291, OQ306534, and OQ383346, respectively. The results of the BLAST searches of the recovered isolate (GKH-2) sequences with the related *N. dimidiatum* sequences were shown in the Table S1. The selected sequences for phylogenetic analysis were presented in Table 1. The maximum likelihood phylogenetic tree was generated for ITS-TEF (Fig. 2), ITS-LSU (Fig. 3) and ITS-LSU-TEF (Fig. 4) using the Tamura 3-parameter and rate among sites has invariant sites (T92 + I), Kimura 2-parameter with distribute Gamma category 2 (K2 + G), and Tamura 3-parameter with distribute Gamma category 2 (T92 + G), respectively. These parameters were the best model that was suggested by MEGA software. To generate ITS-TEF and ITS-LSU-TEF phylogenetic trees, the sequence of *tef1-α* genomic region for *N. orchidacearum* and *N. oculus* was not available in GenBank as well as the LSU genomic region of *N. hylocereum* for generating ITS-LSU and ITS-LSU-TEF phylogenetic trees, so these taxa were omitted from the further analyses. The maximum parsimony tree was also built for the investigated sequences (Fig. S1, S2 and S3) and the tree topology of the two phylogenetic trees for each concatenated sequence were compared. As expected, they were similar, indicating the accuracy of the phylogenetic study. In the phylogenetic trees, the isolate GKH-2 of the present study was placed in a clade along with the *Neoscytalidium* isolates from Turkey with 83% bootstrap support in ITS-LSU phylogenetic tree, with 84% bootstrap support in ITS-LSU-TEF phylogenetic tree and with 56% in ITS-TEF phylogenetic tree, and then it was identified as *N. dimidiatum*. This isolate was deposited in the microbial culture collection of Agricultural Biotechnology Research Institute of Iran Culture Collection, Karaj, Iran, with the accession number of ABRIICC 10,347.

**Table 1:** Sequence data used in the phylogenetic analyses

Species	Isolate	Accession no		
		ITS	LSU	*tef1-α*
*Neoscytalidium dimidiatum*	Kale4-C	MK788362.1	MK803393.2	MK803351.1
	Arp2-D	MK813852.1	MK813853.2	MK816355.1
	CBS 145.78	KF531816.1	DQ377922.1	KF531795.1
	CBS 251.49	KF531819.1	DQ377923.1	KF531797.1
	CBS 499.66	KF531820.1	DQ377925.1	KF531798.1
	**GKH-2**	**MZ047291**	**OQ306534**	**OQ383346**
*Neoscytalidium novaehollandiae*	CBS122071 (CMW 26,170)	EF585540.1	KF766374.1	EF585580.1
	WAC12688	EF585542.1	EF585549.1	EF585575.1
	WAC12691	EF585543.1	EF585548.1	EF585574.1
	NeNo1	KY499712.1	MH899579.1	MF662595.1
	NeNo2	KY499713.1	MH899580.1	MF662596.1
*Neoscytalidium orchidacearum*	CMU287	KY933091.1	KY933092.1	–
	MFLUCC 12-0533	KU179865.1	KU179864.1	–
*Neoscytalidium* sp. (*N. oculus*)	IOM325287	MG764431.1	MG764432.1	–
*Neoscytalidium hylocereum*	PSU-HP01 TSU-HP01 TSU-HP02	LC590859.1 LC590860.1 LC590861.1	– – –	LC590862.1 LC590863.1 LC590864.1
*Neofusicoccum hongkongense*	CERC 2967	KX278050.1	MF410094.1	KX278155.1
	CERC 2968	KX278051.1	MF410095.1	KX278156.1
*Neofusicoccum sinoeucalypti*	CERC 3415	KX278063.1	MF410107.1	KX278168.1
	CERC 2005	KX278061.1	MF410105.1	KX278166.1
*Botryosphaeria wangensis*	CERC 2298	KX278002.1	MF410044.1	KX278107.1
	CERC 2299	KX278003.1	MF410045.1	KX278108.1
*Botryosphaeria qingyuanensis*	CERC 2946	KX278000.1	MF410042.1	KX278105.1
	CERC 2947	KX278001.1	MF410043.1	KX278106.1
*Phyllosticta phoenicis*	CPC:39164	MW883442.1	MW883833.1	MW890098.1

The sequences of the present study are in boldface

**Figure 5:**
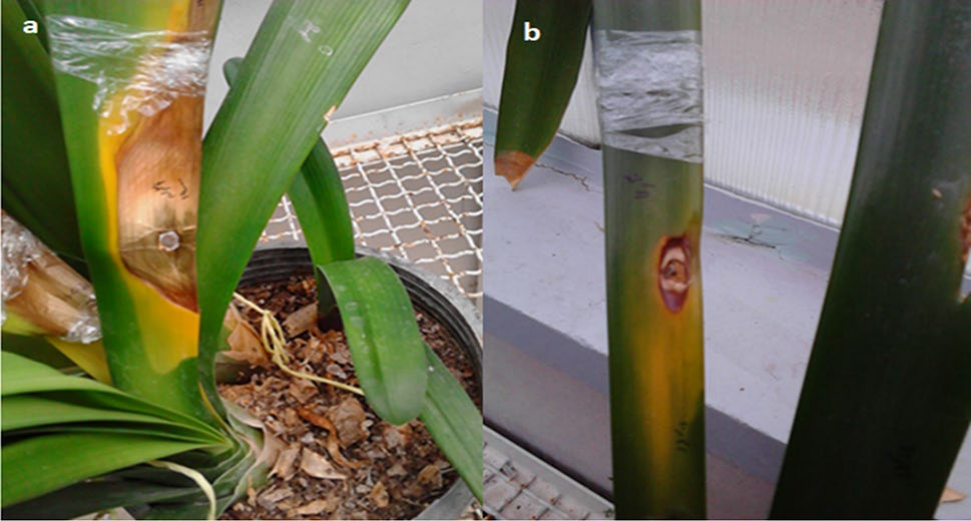
Pathogenicity of *Neoscytalidium dimidiatum* isolate GKH-2 (30–32 ℃; natural day and night light conditions): (**a**) on *C. miniata* 14 dai and (**b**) on *C. nobilis* 27 dai.

In the pathogenicity test, symptom of the disease appeared five days after the plant inoculation in all inoculated sites in *C. miniata* leaves in 30–32 ℃, and in the same temperature condition, the symptom in *C. nobilis* leaves appeared 23 days after the plant inoculation in some of the inoculated sites. At the temperature condition of 26–28 ℃, the symptom of the disease was observed 16 days after the inoculation only in one inoculated site in *C. miniata*, whereas the symptoms were appeared on *C. nobilis* 31 days after the inoculation in one inoculated site. The control plants remained symptomless until the end of the experiment. The blight symptom initiated from the wounded and inoculated sites as a brown-rotted lesions, and the areas around the lesions were chlorotic (Fig. 5). As the disease developed, the brown region at the center of the spot became necrotic. Eventually, the disease led the leaf to die. The inoculated fungus was reisolated from these newly produced symptoms and then it was identified as *N. dimidiatum*, fulfilling the Koch’s postulates.

## Discussion

The *N. dimidiatum* (GKH-2) was isolated from the leaf blight symptom of *C. miniata*. It was identified based on the morphological and molecular characterization. The phylogenetic analysis based on the three genomic regions showed that the isolate GKH-2 belongs to the clade that the *N. dimidiatum* isolates from Turkey were located, and these isolates separated from the clade that accommodates the other *Neoscytalidium* isolates. These two clades were separated with 99% (in the maximum likelihood phylogenetic tree) and 100% (in the maximum parsimony phylogenetic tree) bootstrap value. The separation of the *Neoscytalidium* isolates into two clades, which places isolates from Turkey in one clade independently from other *Neoscytalidium* isolates was in concordance with Zhang et al.^10^ and Güney et al.^17,62^. It is likely that the genus *Neoscytalidium* might be polyphyletic. According to the morphological investigation, isolate GKH-2 was very similar to the *N. dimidiatum* described by Crous et al.^4^, but it produced swollen hyphae rarely and two-septate arthroconidia was also common. The pycnidium formation in the isolate GKH-2 was not observed. Previously, the *N. oculus* isolate IOM325287 was introduced as a new *Neoscytalidium* species based on the phylogenetic analysis as well as lack of pycnidium formation^8^. Regarding to several studies, lack of pycnidium formation is dependent on various factors, such as temperature^1,9,62–66^, and considering this character as a key feature in the description of the species would be inaccurate. Recently, in the phylogenetic study of the *Neoscytalidium* species, it is observed that all species of *Neoscytalidium* are placed in the same clade, which this clade was named as *N. dimidiatum*, except for the isolates from Turkey that located in another clade, which this clade was named as *Neoscytalidium* sp. It has been concluded that the *N. dimidiatum* is the only species in the genus *Neoscytalidium*, so the other species were transferred to this species^10^. For the identification and introduction of *Neoscytalidium* species, all aspects should be considered and polyphasic studies are necessary. Attention to the morphological characteristics and ecological niches such as host along with the phylogenetic studies, can lead to better understanding of the species concept in the genus *Neoscytalidium.* According to the previous studies, *N. novaehollandiae* is distinguished from *N. dimidiatum* by producing two types of conidia, as well as by phylogenetic features^5^. The *N. orchidacearum* produces hyaline conidia, and it was found only on orchid plants in Thailand so far. Considering these features along with phylogenetic characteristics, this species can be separated from *N. dimidiatum* and *N. novaehollandiae*^3,25^. As the Zhang et al.^10^ have mentioned, this opinion that the genus *Neoscytalidium* is monotypic needs further studies, especially in the aspect of genetic population.

Regarding to *N. orchidacearum* and *N. hylocereum*, it seems that host plant has an important role in the evolution and speciation of the genus *Neoscytalidium*. Recently, two isolates of *N. dimidiatum* (isolates Keningau and Keningau02) have been reported causing stem canker on *Selenicereus megalanthus* in Malaysia^67^. These isolates were separated from *N. dimidiatum* and *N. novaehollandiae* based on the ITS, *tef1-α* and *tub* genomic regions in the phylogenetic tree as well as from the *N. hylocereum* isolates of the Wonglom et al.^12^ study. It seems that the isolates Keningau and Keningau02 might be the different species from *N. dimidiatum* and it is suggested that they have to be examined in detail.

Although *N. dimidiatum* almost infects the woody plants, but there are some reports that this species causes disease on leafy plants. The *N. dimidiatum* has been reported as the leaf blight causal agent on white spider lily (*Hymenocallis littoralis*) in Malaysia, Cattleya × hybrid in Taiwan, *Sansevieria trifasciata* in Brazil, *Origanum onites* and *Melissa officinalis* in Turkey^18,19,22,68,69^. Based on our observation of the surveyed greenhouse, about 16 percent of the growing *Clivia* plants had the leaf blight symptom, similar to the sampled symptom, showing the importance of the emerging leaf blight disease on *Clivia* plants caused by *N. dimidiatum*. We did not investigate the special favorable conditions for the infection of the *Clivia* plants by the pathogen, except two temperature ranges 26–28 ℃ and 30–32 ℃. But, like any other plant disease, the leaf blight disease occurrence on *Clivia* might be affected by any kind of stresses such as temperature and water stresses. The effect of temperature on pathogenicity of the recovered isolate was investigated in this study. The temperature ranging from 26 to 28 ℃ could not stimulate the pathogenicity of *N. dimidiatum* isolate GKH-2. The temperature ranging from 30 to 32 ℃ was optimal to isolate GKH-2 to cause disease on *Clivia* leaves. This obtained result is in concordance to the fact that *N. dimidiatum* is a thermophilic fungus and tends to cause diseases in the tropical and subtropical regions^14,62^. The optimal temperature is an environmental factor that required to induce the pathogenicity and development of the disease by *Neoscytalidium* species. It has been demonstrated that the temperature is the effective factor for disease onset^70^. It has also been mentioned that the temperature is a critical factor for successful infection and disease severity caused by pythiaceous fungi^71^. It has demonstrated that the highest fig fruit colonization by *N. dimidiatum* occur at 30 ℃ and the mycelium of *N. dimidiatum* can not develop at 10 and 15 °C^72^.

According to the results of the pathogenicity test, the isolate GKH-2 was pathogenic on both *C. miniata* and *C. nobilis*. Regarding to the time after inoculation for symptom development on *C. miniata* and the severity of the symptoms, and in comparison with *C. nobilis*, it seems that the isolate GKH-2 was more pathogenic on *C. miniata*. *Neoscytalidium dimidiatum* is known as an opportunistic fungus that penetrates through the wounds and causes diseases under the stress conditions^6,62^. However, It has been mentioned that this fungus can also cause diseases in healthy hosts^13,62^. To our best of knowledge, this is the first report of *N. dimidiatum* causing leaf blight disease on *C. miniata* and *C. nobilis*, worldwide.

### Supplementary Information

Supplementary Information.

## Data Availability

Most of the datasets generated during and/or analyzed during the current study are available in the manuscript. The newly generated sequences are deposited in the GenBank (MZ047291, OQ306534 and OQ383346).
