# Supplementary material for: First report of Neoscytalidium dimidiatum as the causal agent of leaf blight on Clivia miniata
Source: Sci Rep. 2023 Sep 26;13:16110. doi: 10.1038/s41598-023-43144-4 (PMC10522640; doi:10.1038/s41598-023-43144-4)
Supplement: Supplementary file 1 — Supplementary Information. [file 41598_2023_43144_MOESM1_ESM.docx]

**Supplementary Information**

| **Isolate** | **Query coverage** | | | **Identity** | | |
| --- | --- | --- | --- | --- | --- | --- |
|  | **ITS** | **LSU** | ***tef1-α*** | **ITS** | **LSU** | ***tef1-α*** |
| Arp2-D | 97% | 63% | 87% | 99.63% | 100% | 100% |
| Kale4-C | 97% | 63% | 87% | 99.63% | 100% | 100 % |
| CBS 145.78 | 99% | 94% | 87% | 99.09% | 99.77% | 100% |
| CBS 251.49 | 98% | 94% | 87% | 99.09% | 99.77% | 100% |
| CBS 499.66 | 98% | 94% | 87% | 98.91% | 99.77% | 98.46% |

**Table S1.** The results of query BLAST search in NCBI. All isolates belong to *Neoscytalidium dimidiatum*.


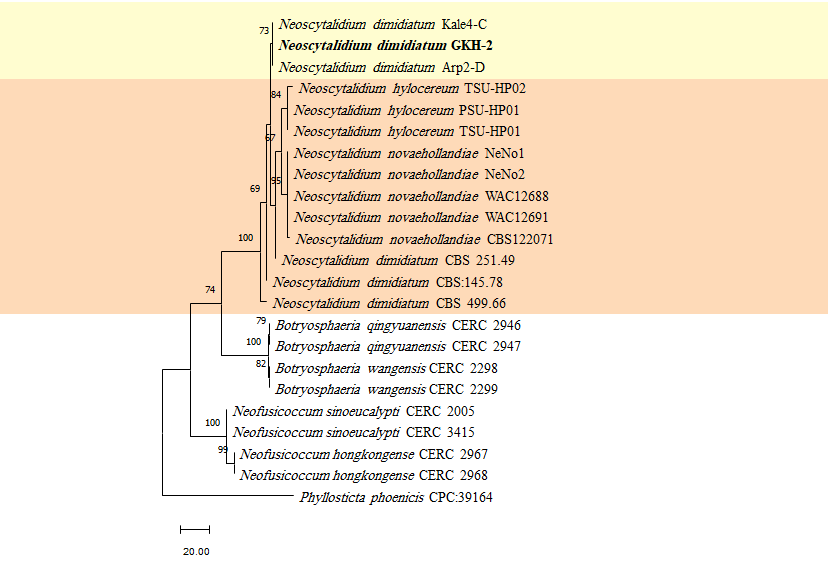
**Figure S1.** Maximum parsimony phylogenetic tree of concatenated ITS and *tef1-α* sequences, generated by MEGA ver. 11 software. *Phyllosticta phoenicis* was used as an outgroup taxon. The recovered fungal isolate in the present study was shown in boldface. The bootstrap values more than 50% have been presented on the branches.

**
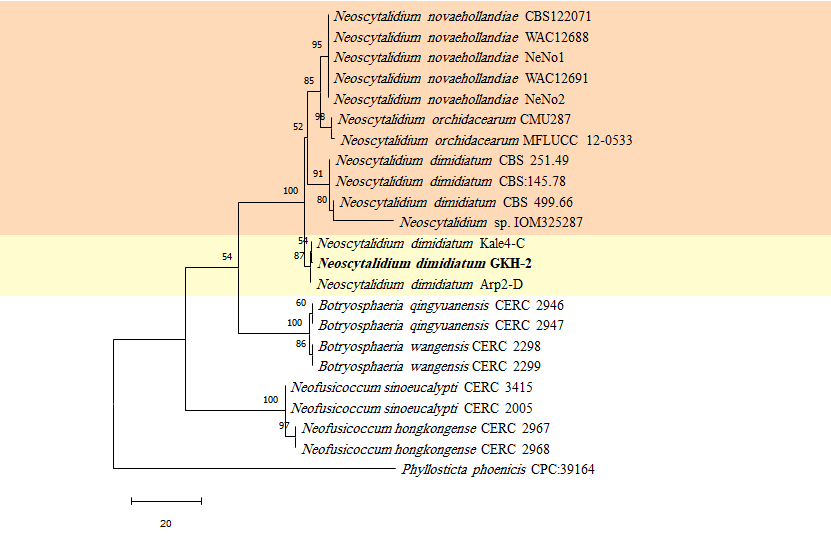
**

**Figure S2.** Maximum parsimony phylogenetic tree of concatenated ITS and LSU sequences, generated by MEGA ver. 11 software. *Phyllosticta phoenicis* was used as an outgroup taxon. The recovered fungal isolate in the present study was shown in boldface. The bootstrap values more than 50% have been presented on the branches.


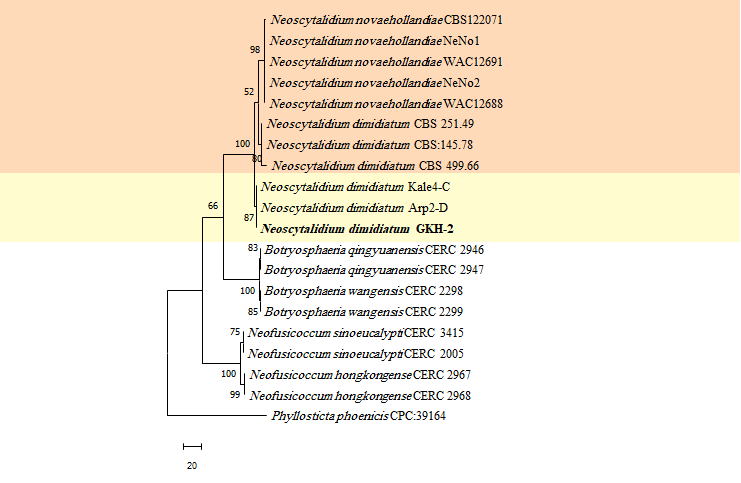
**Figure S3.** Maximum Parsimony phylogenetic tree of concatenated ITS, LSU and *tef1-α* sequences, generated by MEGA ver. 11 software. *Phyllosticta phoenicis* was used as an outgroup taxon. The recovered fungal isolate in the present study was shown in boldface. The bootstrap values more than 50% have been presented on the branches.
